# Complete Genome Sequence of *Mycolicibacterium* sp. Strain NIID-NTM18, Isolated from Cardiac Implantable Electronic Device Infection and Most Similar to Mycolicibacterium litorale

**DOI:** 10.1128/MRA.00830-20

**Published:** 2020-09-03

**Authors:** Hanako Fukano, Hiroshi Miyama, Seiji Takatsuki, Aki Hirabayashi, Masato Suzuki, Mitsunori Yoshida, Yoshifumi Uwamino, Naoki Hasegawa, Yoshihiko Hoshino

**Affiliations:** a Department of Mycobacteriology, Leprosy Research Center, National Institute of Infectious Diseases, Tokyo, Japan; b Department of Cardiology, Keio University School of Medicine, Tokyo, Japan; c Antimicrobial Resistant Research Center, National Institute of Infectious Diseases, Tokyo, Japan; d Department of Laboratory Medicine, Keio University School of Medicine, Tokyo, Japan; e Center for Infectious Diseases and Infection Control, Keio University School of Medicine, Tokyo, Japan; University of Maryland School of Medicine

## Abstract

Mycolicibacterium litorale is a rapidly growing mycobacterial organism with unknown pathogenic features. Here, we report the complete genome sequence of *Mycolicibacterium* sp. strain NIID-NTM18, which was isolated from a cardiac implantable electronic device infection and which is most similar to *M. litorale*. This sequence will provide essential information for future studies of the pathogenicity of these mycobacteria.

## ANNOUNCEMENT

Nontuberculous mycobacteria (NTMs) are an important cause of human infectious diseases and exist ubiquitously in the environment (1). NTM infections resulting from cardiac implantable electronic devices (CIEDs) are uncommon; however, such infections are likely to be reported, and most (70%) of these infections result from rapidly growing nontuberculous mycobacteria (RGMs) (2). Many device/catheter-related RGM infections have been recorded to date (3–5).

Mycolicibacterium litorale is an RGM species that was originally isolated from an environmental soil sample in Haikou, China. Our previous study showed that this species could cause CIED infection (6, 7). Here, we report the complete genome sequence of *Mycolicibacterium* sp. strain NIID-NTM18, which is most similar to *M. litorale*. The findings are expected to improve our understanding of the pathogenic features of this strain.

Strain NIID-NTM18 was isolated from catheter lead pus from an implanted pacemaker in Japan (6). A single clone was isolated on Middlebrook 7H10 agar supplemented with 10% oleic acid-albumin-dextrose-catalase (OADC) enrichment (Becton Dickinson, USA) at 37°C. Genomic DNA for short-read Illumina sequencing was extracted using a NucleoSpin plant II kit (Macherey-Nagel, Germany), and that for long-read sequencing was extracted using a standard phenol-chloroform extraction technique. A paired-end sequencing library was prepared using a Nextera XT DNA sample prep kit (Illumina, Inc., San Diego, CA) according to the manufacturer’s instructions. Illumina paired-end (2 × 150-bp) reads (1,230,040 reads; 61× coverage) were obtained using the NovaSeq 6000 system (Illumina) and assessed by FastQC (Galaxy version 0.72+galaxy1) (8). Long-read sequence reads were obtained with a MinION instrument (Oxford Nanopore Technologies, Oxford, UK). Sequencing was performed with an SQK-RAD004 rapid barcoding sequencing library kit in accordance with the manufacturer’s protocol. The library was loaded onto a SpotON Mk I (R9.4) flow cell and sequenced using MinKNOW version 19.12.2. Raw sequence data (FAST5 format) were base called using Guppy version 3.4.1 software, and a total of 54,843 reads (*N*_50_, 16,022 bp; coverage, 55×) were generated. Hybrid assembly was performed by using Unicycler with default settings (Galaxy, version 0.4.8.0), and one contig was circular as called by Unicycler (5,634,149 bp) (9). The resulting contig sequence was annotated using DFAST with default settings (10).

The chromosome of strain NIID-NTM18 was 5,634,149 bp (G+C content, 68.9%). The numbers of predicted coding sequences, rRNAs, and tRNAs in the genome were 5,480, 6, and 55, respectively.

The sequences of five complete housekeeping genes (16S rRNA, *hsp65*, *rpoB*, *recA*, and *sodA* genes) showed the highest similarity with those of *M. litorale* type strain JCM17423 by BLAST search. The average nucleotide identity (ANI) compared with the nearly complete genome sequence data of *M. litorale* JCM17423^T^ (GenBank accession number NZ_AP022586.1) was calculated to be 86.83% using JSpeciesWS (11).

The complete genome sequence of *Mycolicibacterium* sp. strain NIID-NTM18, which was isolated from a CIED infection and is most similar to *M. litorale*, provides essential data for future taxonomic analysis and studies of the pathogenicity of mycobacteria.

### Data availability.

The genome sequence and annotations of *Mycolicibacterium* sp. strain NIID-NTM18 were deposited in DDBJ/EMBL/GenBank under accession number AP023287. The raw sequence data are available in the DDBJ Sequence Read Archive under accession numbers DRX226425 and DRX226426.
